# Persistent spatial structuring of coastal ocean acidification in the California Current System

**DOI:** 10.1038/s41598-017-02777-y

**Published:** 2017-05-31

**Authors:** F. Chan, J. A. Barth, C. A. Blanchette, R. H. Byrne, F. Chavez, O. Cheriton, R. A. Feely, G. Friederich, B. Gaylord, T. Gouhier, S. Hacker, T. Hill, G. Hofmann, M. A. McManus, B. A. Menge, K. J. Nielsen, A. Russell, E. Sanford, J. Sevadjian, L. Washburn

**Affiliations:** 1 Department of Integrative Biology, Oregon State University, Corvallis, OR USA; 2 College of Earth, Ocean, and Atmospheric Sciences, Oregon State University, Corvallis, OR USA; 3 Marine Science Institute, University of California Santa Barbara, Santa Barbara, CA USA; 4 College of Marine Science, University of South Florida, St. Petersburg, FL USA; 5 Monterey Bay Aquarium Research Institute, Moss Landing, CA USA; 6 Pacific Coastal and Marine Science Center, United States Geological Survey, Santa Cruz, CA USA; 7 Pacific Marine Environmental Laboratory, National Oceanic and Atmospheric Administration, Seattle, WA USA; 8 Bodega Marine Laboratory, University of California Davis, Bodega Bay, CA USA; 9 Department of Evolution and Ecology, University of California Davis, Davis, CA USA; 10 Marine Science Center, Northeastern University, Nahant, MA USA; 11 Department of Earth and Planetary Sciences, University of California Davis, Davis, CA USA; 12 Department of Ecology, Evolution and Marine Biology, University of California Santa Barbara, Santa Barbara, CA USA; 13 Department of Oceanography, University of Hawaii at Manoa, Honolulu, HI USA; 14 Romberg Tiburon Center for Environmental Studies, San Francisco State University, Tiburon, CA USA; 15 Marine Science Institute and Department of Geography, University of California Santa Barbara, Santa Barbara, CA USA

## Abstract

The near-term progression of ocean acidification (OA) is projected to bring about sharp changes in the chemistry of coastal upwelling ecosystems. The distribution of OA exposure across these early-impact systems, however, is highly uncertain and limits our understanding of whether and how spatial management actions can be deployed to ameliorate future impacts. Through a novel coastal OA observing network, we have uncovered a remarkably persistent spatial mosaic in the penetration of acidified waters into ecologically-important nearshore habitats across 1,000 km of the California Current Large Marine Ecosystem. In the most severe exposure hotspots, suboptimal conditions for calcifying organisms encompassed up to 56% of the summer season, and were accompanied by some of the lowest and most variable pH environments known for the surface ocean. Persistent refuge areas were also found, highlighting new opportunities for local adaptation to address the global challenge of OA in productive coastal systems.

## Introduction

Eastern boundary current upwelling systems such as the California Current Large Marine Ecosystem (CCLME) represent one of the ocean’s most productive biomes, sustaining one-fifth of the world’s fisheries^1^. Broad-scale observations^2^, and models^3^ have highlighted the biogeochemical sensitivity of the CCLME to the rapid progression of ocean acidification (OA). This susceptibility reflects the central role of upwelling currents in connecting coastal waters with the dissolved inorganic carbon (DIC)-rich ocean interior, and the high potential for active carbon remineralization over productive continental shelves. Against this naturally-elevated DIC baseline, present day anthropogenic CO_2_ burdens upwards of 60 μmol kg^−1^ have already resulted in declines of up to 0.12 and 0.5 in pH and **Ω**_arag_, respectively^3, 4^. Within the CCLME, model and broad-scale cruise surveys have further identified the nearshore as the region most strongly affected by present, and likely future, expression of OA^2–4^ and is where the most frequent occurrences of severe shell dissolution in planktonic pteropods can already be found^5^.

The nearshore waters (within 10 km of coast) of the CCLME contain crucial but vulnerable ecological and fishery habitats, encompassing nearly all U. S. West Coast kelp forests and marine reserves, as well as biologically diverse intertidal habitats where calcifying organisms often dominate ecological structure and function^6^. While science and management decisions such as the placement of marine reserves in the CCLME have been informed by important spatial patterning in ocean circulation, population connectivity, biodiversity, and climate vulnerability^7–9^, basic information on whether the CCLME faces uniform or heterogeneous exposure to the progression of OA is lacking. For coastal systems most at risk from OA, this limitation impairs our understanding of how biological and socio-economic vulnerability^10^ are distributed, and whether spatial management tools can be applied to ameliorate local impacts of globally-driven changes in ocean chemistry^11^. To resolve this uncertainty, we implemented an ocean observing network consisting of custom-designed pH sensors deployed in rocky intertidal habitats (see supplementary materials for methods) to provide the first high-frequency, multi-year view of near-shore OA progression across 1,000 km of the CCLME.

## Results

**Figure 1:**
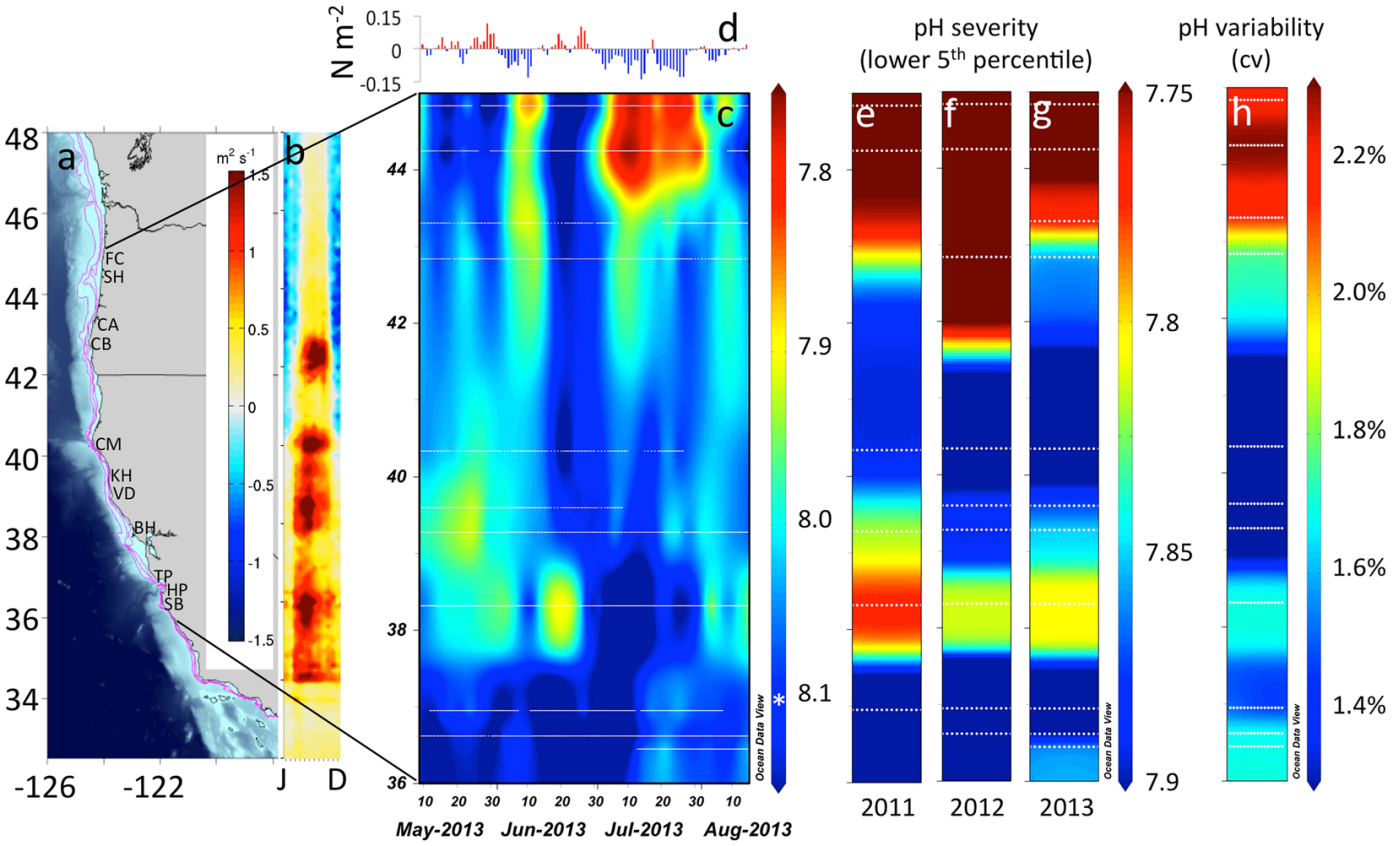
(**a**) Intertidal pH_total_ variation across the CCLME study domain of contrasting shelf topography as delineated by the 75 m, 100 m, 200 m isobaths (magenta), and (**b**) wind-driven cross-shelf surface transport (m^2^ s^−1^)(see scale inset in a). Map was generated from the ETOPO1 dataset^42^ in Matlab v8.2 (http://www.mathworks.com/products/matlab). (**c**) Variation in pH at the event-scale during part of the 2013 upwelling season with asterisk denoting global mean surface pH^12^ and (**d**) accompanying daily wind stress (N m^−2^) at 44.65°N, (**e**–**g**) severity of low pH exposure (lower 5^th^ percentile) across three years of deployment and (**h**) pH variability. White dotted lines in pH panels denote station locations.

**Figure 2:**
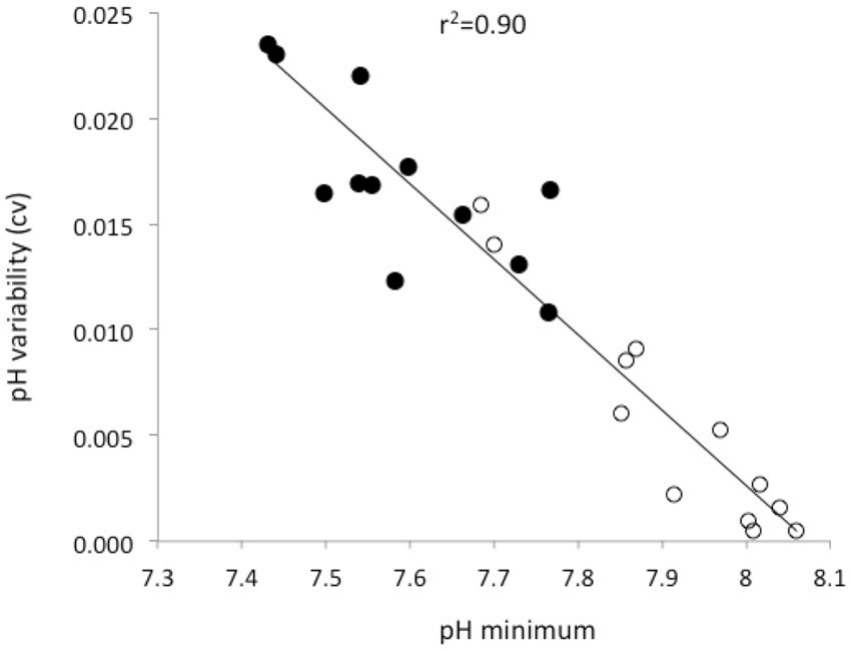
Increase in pH variability (coefficient of variability) as a function of pH minimum across surface ocean datasets from present study (solid circles) and from a cross-biome survey^13^ (open circles) that includes representation from tropical, temperate, and polar surface ocean sites. Three sites that are strongly influenced by unique local conditions (an estuary, a volcanic CO_2_ vent and a groundwater discharge site) are excluded.

Across the CCLME, we observed near-shore pH that fell well below current global mean surface ocean pH of 8.1^12^ (Fig. 1). Minimum pH reached as low as 7.43 at the most acidified site, and up to 18% of recorded values fell below 7.8 during the upwelling season (Table S1). These minima are among the lowest reported to date for the surface ocean^13^ and match levels not projected for the global surface ocean until atmospheric CO_2_ exceeds 850 ppm^14^. Exposure regimes to OA were also highly dynamic, exhibiting strong diel as well as event (2 to 10 day-), and intra-seasonal (~15 to 40 day) -scale variability. Daily pH ranges of up to 0.8 units were measured, as were rapid rates of change of up to 0.3 pH units per hour. Our findings indicate that coastal organisms in the CCLME face not only some of the lowest, but also some of the most dynamic pH environments currently known for surface marine systems (Fig. 2). Because variable pH exposure can affect organism response to OA^15, 16^, low and variable pH can further act as linked stressors. Future biological impacts experiments should consider the strong but predictable co-variation between pH minimum and variability as key elements of realistic exposure conditions.

Despite high-frequency temporal variability in intertidal pH, the deployment of sensors across years revealed distinct geographic structuring to low pH exposure. Coastal OA emerges as a spatial mosaic where a regional “hotspot” of lowest and most variable pH exposure (Fig. 1) recurs each year in the northern CCLME. The distribution of OA risk however, did not follow a simple latitudinal trend nor did it vary spatially with broad-scale patterns of upwelling wind-stress (Fig. 1b). For example, at Cape Mendocino (CM, 40.34°N), a prominent coastal headland where upwelling wind stress is accentuated, pH never fell below 7.76 while a second low pH region is evident at Bodega Head (BH, 38.32°N). This pattern of spatial structuring was highly persistent across three years of observations that spanned periods of neutral and negative ENSO activity.

**Figure 3:**
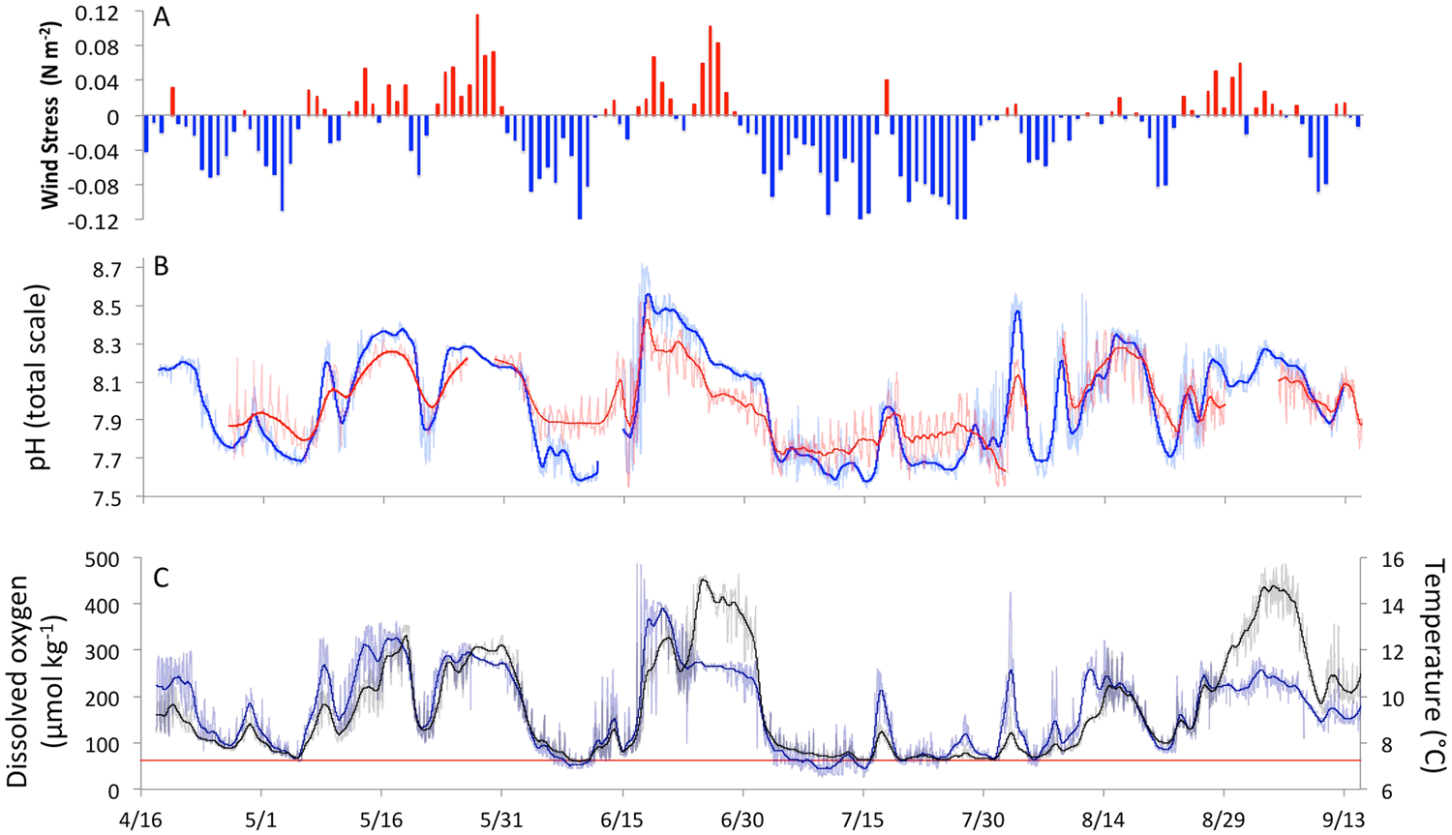
(**a**) 2013 wind forcing as indexed by along-shore wind stress at 44.65N, (**b**) intertidal pH (red) and inner-shelf pH (blue) at 44.25°N, (**c**) temperature (black) and dissolved oxygen (blue) from moorings deployed directly offshore (15 m water depth). Red line in (**c**) denotes hypoxia threshold of 65 μmol kg^−1^. Bold lines in (**b**,**c**) denote low-pass filtered (40 h window LOESS filter) time-series.

**Figure 4:**
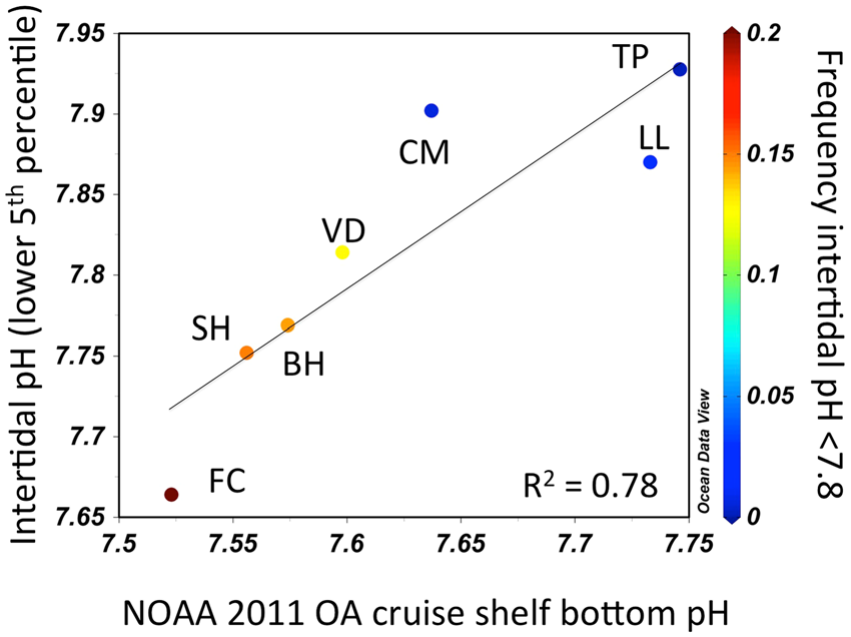
Correlation between point-in-time measurement of off-shore (water depth range of 60 to 125 m) near-bottom pH in 2011 from the (x-axis) and intertidal measurements of seasonal severity (lower 5^th^ percentile, left y-axis) and frequency (% observations with pH < 7.8, denoted by symbol color and color bar) of low pH conditions. For station locations, refer to Table S1.

Our results also indicate that intertidal pH is not simply a product of highly localized surf-zone processes. Intertidal pH fluctuated in concert with both upwelling winds and shelf pH measured directly offshore (Fig. 3, Figs S9 and S10), declining sharply with the onset of upwelling-favorable wind events that transport cold, low-pH waters from depth to the coast, and rising with downwelling events that bring warm, high-pH surface waters shoreward. At the spatial resolution of our network, such upwelling events can result in low-pH episodes that extend across 200 km of coastline (Fig. 1). In 2011, the passage of a NOAA OA survey cruise^17^ through the study region allowed us to further evaluate the broader-scale origin of the near-shore spatial pH mosaic. One-time ship-based pH profiles show a surprisingly strong correspondence between both the severity and frequency of low pH events encountered in the intertidal sensor network, and pH measured from continental shelf stations at depth (60–125 m depth) (Fig. 4, Fig. S8a). Correspondence between offshore surface pH values and variation in high pH events can also be seen (Fig. S8b). In conjunction with previously established distribution of carbonate chemistry changes offshore^3, 5^, our findings indicate that OA has already progressed to be a perturbation that extends across the continental shelf and into the land-sea interface of the CCLME (Fig. S9).

Low dissolved oxygen (DO) has documented impacts on marine life in the CCLME^18^, can accentuate the vulnerability of organisms to OA^19^, and is a key issue of management concern^20^. We found that the most severe low pH events were consistently accompanied by the onset of hypoxia. Coastal organisms in the CCLME thus face an OA exposure regime that is not only highly temporally dynamic but also intimately tied to the co-occurrence of low DO stress. The progression of OA is intrinsically linked to the rate of ocean CO_2_ uptake^1, 3^. The coupling between surf-zone pH, atmospheric forcing, and shelf pH further suggests that climate-dependent processes such as intensification of upwelling wind stress^21, 22^ and ocean deoxygenation (when coupled to carbon remineralization)^23^ can modulate and potentially accelerate the rate of OA progression in the near-shore waters of eastern boundary current upwelling systems^24^.

**Figure 5:**
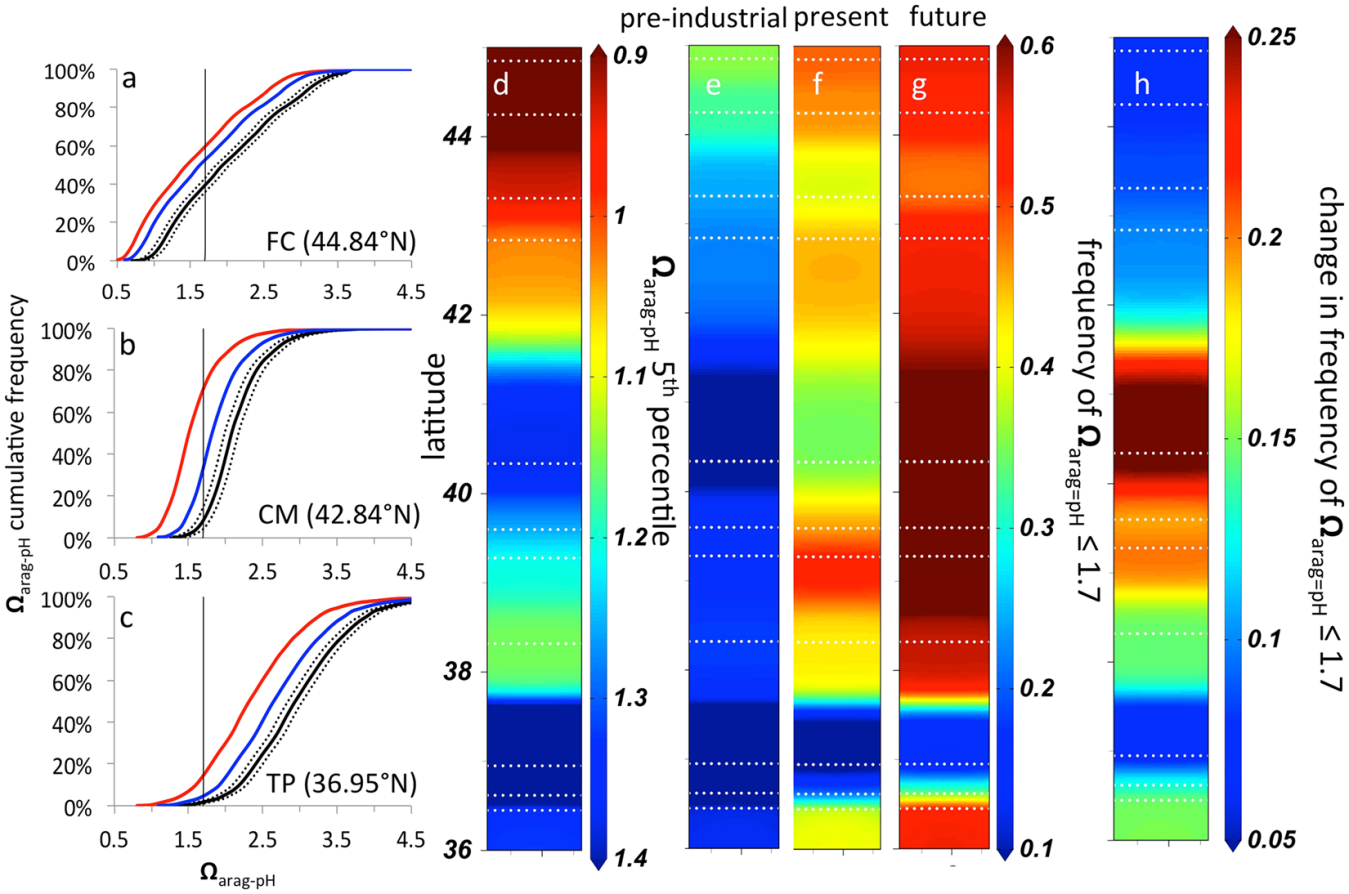
Changes in cumulative frequency of low aragonite saturation state events for three example sites (**a**–**c**) FC = Fogarty Creek, CM = Cape Mendocino, TP = Terrace Point) from pre-industrial (black), to present observations (blue), to future conditions (red). Black dotted lines represent ±3 s.d. window for estimates of pre-industrial states. Future conditions reflect the mean of an additional 22 μmol kg^−1^ of DIC that accompanies the arrival of water equilibrated with present-day 400 ppm atmosphere to the CCLME. Vertical lines in (**a–c**) denote Ω_arag_ values of 1.7. Latitudinal patterns are shown for current lower 5^th^ percentile Ω_arag-pH_ (**d**), and projected past (**e**) present (**f**) and projected future (**g**) frequencies of Ω_arag-pH_ ≤ 1.7, and frequency changes in Ω_arag-pH_ ≤ 1.7 between the present and future projections (**h**).

While pH is a fundamental property of the carbonate system, OA can also affect organism performance through *p*CO_2_, HCO_3_^−^, and/or CO_3_^2–^dependent pathways^25–27^. In the CCLME, aragonite saturation state (**Ω**_arag_) has emerged as a highly predictive parameter for linking carbonate chemistry change to biological impacts in a number of important taxa^5, 27, 28^. Calculation of **Ω**_arag_, however, requires at least one additional carbonate system parameter and ideally total alkalinity (A_T_) or DIC, as pH and *p*CO_2_ covary strongly. *In-situ* sensors for DIC or A_T_ are not currently available for continuous surf-zone deployment and proxy approaches for estimating **Ω**_arag_ from DO^29^ have not been verified for surface near-shore waters. We derived an estimate of **Ω**_arag_ (Fig. 5, denoted as **Ω**_arag-pH_) using measured pH and temperature, together with mean values of salinity and alkalinity that takes advantage of the strong covariation between pH and **Ω**_arag_ in the system. Values of **Ω**_arag-pH_ and **Ω**_arag_ calculated from discrete bottle samples showed high agreement (r^2^ = 0.99) (Fig. S1). Application of **Ω**_arag-pH_ to regional and global sets (Fig. S2) further suggests that **Ω**_arag-pH_, when constrained by discrete samples, can provide a robust approximation of **Ω**_arag_, particularly for low (<2) **Ω**_arag_ values where concern is greatest.

Our estimates of Ω_arag-pH_ indicate that conditions corrosive to aragonite already blanket nearshore coastal habitats across large portions of the CCLME (Fig. 5). Corrosive conditions were evident for all sites. At one of the most acidified sites (SH, 44.25°N), up to 16% percent of Ω_arag-pH_ fell below 1. Additionally, studies to date indicate that biological impacts can occur well before thermodynamic solubility is reached^27, 30^. Across the network, up to 63% of estimates of **Ω**_arag-pH_ already fall below 1.7, a threshold associated with commercial production failures for larval oysters in the system^28^.

While low Ω_arag_ is expected in DIC-rich upwelling systems, our calculations suggest that frequency of low **Ω**_arag-pH_ exposure has increased due to the contributions of anthropogenic DIC (DIC_ant_). Following approaches for calculating DIC_ant_ as a function of density in the CCLME^3, 4^, we estimate that water upwelled to the shore holds a mean DIC_ant_ burden of 37 (±3.5, s.d.) μmol kg^−1^. This DIC_ant_ contribution is equivalent to a 0.3 to 0.4 decrease in mean **Ω**_arag_ since the pre-industrial era. Subtracting this burden from our observed values, the frequency of **Ω**_arag_ < 1.7 events across sites declines by as much as 39% (Fig. 4, Table S1). Our estimate of the mean DIC_ant_ burden reflects the difference between equilibration with a pre-industrial (280 ppm) atmosphere and water masses that on average last equilibrated with a 1980’s (350 ppm) atmosphere in the western Pacific, the source region for water upwelled in the CCLME. Water forming and equilibrating with today’s 400 ppm atmosphere will have a mean DIC_ant_ burden of ~56 μmol kg^−1^. When this water reaches the CCLME, the frequency of **Ω**_arag_ < 1.7 events at the CM site (40.34°N) in northern California will rise to 61%, an 81% increase from current exposure and 14.5-fold increase over pre-industrial estimates (Fig. 5). We further find that the progression of OA will not be uniform. Sites in northern California that currently experience only moderate exposure to **Ω**_arag_ < 1.7 events, but have a high percentage of exposures near that threshold are projected to be most impacted by OA in the future. These results highlight both the scale and geography of unavoidable chemistry changes that coastal habitats will face in the coming decades.

## Discussion

The expression of OA as a persistent spatial mosaic has important ecological, evolutionary and management implications. Populations in regions of persistent low pH might be locally adapted to OA^31, 32^. As dispersal sources for stress-resistant genotypes, these populations would play a critical role in supporting biological resilience to increasing OA stress over broader spatial scales. Conversely, regions currently facing moderate pH exposure can serve as near-term refuges, particularly for organisms with limited adaptive capacity for coping with the pace and scope of carbonate chemistry changes. While our efforts have identified strong regional separation in OA exposure, further observations will be needed to define the long-term stability of the coastal OA mosaic particularly as the CCLME transitions through additional states of climate variability and change. Expansion of paired shelf and nearshore observations will also be important for further testing the strength of cross-shelf coupling in OA exposure. Finer-scale features such as upwelling centers and river plumes are important spatial attributes of eastern boundary current systems^33^. At even finer-scales, inner-shelf circulation processes that affects the efficiency of cross-shelf flows^34, 35^, and benthic macrophytes that directly modulate carbonate chemistry^36, 37^ may weaken cross-shelf coupling and give rise to further spatial patterning in OA exposure at scales that lie beyond the resolution of our study. Resolving the potential for OA to vary predictably over such scales will be important as that knowledge can further advance local socio-economic vulnerability^10^ and integrated ecosystem assessments^38^ that have to date relied on coarse-scale oceanographic estimates to inform local impacts and decisions. Collectively, our emerging understanding of the geography of OA progression across the CCLME highlights key needs and opportunities for local adaptation planning in response to global scale changes in ocean chemistry.

## Materials and Methods

Intertidal pH (total scale) was measured using Durafet®-based (Honeywell Inc.) pH sensors custom-designed for nearshore marine deployment for this project. In 2012, Durafet®-based sensors (Seafet) constructed by Dr. Todd Martz (Scripps Institute of Oceanography) were deployed at 3 additional sites. Depending on year, pH sensors were deployed at 7 to 12 sites (Table S1) during the core upwelling season (April to October). Sensors were secured to the bedrock and serviced and calibrated at 4 to 8 week intervals. Sensors were periodically emergent during low tides and these records were excluded from analyses. Each unit was calibrated directly against TRIS-and/or seawater-based certified reference material (CRM) from Dr. Andrew Dickson’s group (Scripps Institute of Oceanography) or indirectly against seawater CRM-calibrated spectrophotometrically-determined pH samples. This permits the accuracy of each deployment to be traceable to a CRM standard.

At 44.85°N, a mooring (LB-15) was deployed in the inner-shelf (15 m depth) offshore from the FC intertidal pH sensor. The mooring consisted of a Seafet pH sensor at 4 m depth, and an SBE-43 dissolved oxygen (DO) sensor equipped SBE-16+ conductivity and T sensor at 13 m depth. pH sensors were serviced and calibrated at 4-week using the same method described above for the intertidal pH sensors. The DO sensor was calibrated by conducting paired profiles with discrete Winkler DO samples.

Offshore samples from the 2011 National Ocean and Atmospheric Administration (NOAA) Pacific Marine Environmental Laboratory (PMEL) West Coast Ocean Acidification WCOA2011^39^ were analyzed for pH spectrophotometrically at 25°C using purified meta-cresol purple^40, 41^. For each intertidal station in our 2011 deployments, we plotted the deepest (near-bottom) mid-shelf pH value from the nearest cruise station. Upwelling wind stress for 44.65°N in Fig. 1a, was calculated from NOAA buoy 46050 wind records. Ekman transport in Fig. 1b was calculated from the 27 km grid COAMPS model. West stress values from the third pixel west of the coastline were rotated to an along-coast angle and Ekman transport was calculated at each 12-hr model analysis run and averaged to 1-week intervals prior to contouring. Continental shelf width data in Fig. 1a was derived from the ETOPO1 model^42^.

The solubility of carbonate biominerals such as aragonite broadly covaries with pH (Fig. S3). but pH alone provides a poorly constrained estimate of **Ω**_arag_ (Fig. S3) We developed a proxy variable (**Ω**_arag-pH_) that approximates **Ω**_arag_ from pH by taking into account the influence of T, S, and A_T_. From the bottle samples collected adjacent to intertidal sensors, we calculated **Ω**_arag_ directly from either DIC and A_T_, or pH and A_T_, along with measured T, and S. Comparisons between **Ω**_arag_ against **Ω**_arag-pH_ that used only pH, T, and mean values for S (33.5) and A_T_ (2200 μmol kg^−1^) showed very strong agreement (r^2^ = 0.99, **Ω**_arag_ = 1.01* **Ω**_arag-pH_ + 0.01) (Fig. S1). To evaluate the generality of this approach, we applied this same estimation technique to the upper ocean (0 to 400 m depth) values from the North American Carbon Program (NACP) 2007 west coast cruise dataset^2^ and to the Global Ocean Data Analysis Project (GLODAP) database^43^. In both instances, pH_total scale_ (calculated from DIC and A_T_) in conjunction with mean A_T_ (2200 μmol kg^−1^ for NACP, 2300 μmol kg^−1^ for GLODAP) yielded **Ω**_arag-pH_ that agreed strongly with **Ω**_arag_ derived from DIC and A_T_ (Fig. S2). Further details of the uncertainties associated with the use of **Ω**_arag-pH_ can be found in the supplementary materials.

We estimated anthropogenic DIC (DIC_anth_) following a density-based approach^2, 4^. In the Pacific, DIC_anth_ in the upper ocean ranges from approximately 20 to 60 μmol kg^−1^ depending on time since last equilibration with the atmosphere^4^. From observed density, we estimate a mean DIC_anth_ burden of 37 (+/−3.3, S.D.) μmol kg^−1^ for our system. This corresponds to a DIC increase from preindustrial atmosphere of 280 ppm to ~350 ppm at equilibrium, and reflects the higher mean ventilation age of upwelled water. DIC_anth_ can differ spatially and recent analyses suggests that DIC_anth_ in Northern California portion of the CCLME average 15% (+/−5% S.D.) higher than waters in Oregon^17^. We applied this adjustment, differentiating between Oregon and California stations. To estimate future DIC changes, we calculated the DIC increase associated with a rise in atmospheric CO_2_ from 350 ppm to 400 ppm. Across orthogonal combinations of T (8 to 18 °C), S (32 to 34), and A_T_ (2100 to 2300 μmol kg^−1^), an increase from 350 ppm to 400 ppm results in a mean and highly constrained DIC increase of 22 (+/−1.3, S.D.) μmol kg^−1^. To evaluate the effects of DIC_anth_ changes on the distribution of **Ω**_arag_, we calculated the change in **Ω**_arag_ for the subtraction of 37 μmol kg^−1^ and addition of 22 μmol kg^−1^ of DIC. The effects of these DIC changes varied most strongly as a function of starting **Ω**_arag_. With declining **Ω**_arag_, the marginal effects of DIC changes decrease considerably as pH moves further and further away from pK_2_, where absolute changes in [CO_3_^2–^] approach a minimum. Changes in **Ω**_arag_ with respect to changes in DIC are well-constrained across ranges of T and S (Fig. S4). At **Ω**_arag_ = 3, the removal of 37 μmol kg^−1^ DIC increases **Ω**_arag_ by 0.367 and ranges by only +/−0.014. At **Ω**_arag_ = 1, mean decline of 0.280 exhibits a range of only +/−0.009. This constrained behavior permits us to robustly estimate changes in **Ω**_arag_ as a function of initial **Ω**_arag_ from time-series observations.

## Electronic supplementary material

Chan_Supplmentary_Info
