# Supplementary material for: Persistent spatial structuring of coastal ocean acidification in the California Current System
Source: Sci Rep. 2017 May 31;7:2526. doi: 10.1038/s41598-017-02777-y (PMC5451383; doi:10.1038/s41598-017-02777-y)
Supplement: Supplementary file 1 — Chan_Supplmentary_Info [file 41598_2017_2777_MOESM1_ESM.pdf]

Supplementary Materials File for:

Persistent spatial structuring of coastal ocean acidification in the California Current System

F. Chan, J. A. Barth, C. A. Blanchette, R. H. Byrne, F. Chavez, O. Cheriton, R. A. Feely, G. Friederich, B. Gaylord, T. Gouhier, S. Hacker, T. Hill, G. Hofmann, M. A. McManus, B. A. Menge, K. J. Nielsen, A. Russell, E. Sanford, J. Sevadjian, and L. Washburn

Site	Site code	Station Coordinates	start year	end year	pH frequency ≤7.8	pH lower 5th percentile	pH min	pH mean	pH CV	pH # observa tions	Current $\Omega_{arag\text{-}pH}$ frequency ≤1.7	Current $\Omega_{arag\text{-}pH}$ frequency ≤1.0	Pre-industrial $\Omega_{arag\text{-}pH}$ frequency ≤1.7	Pre-industrial $\Omega_{arag\text{-}pH}$ frequency ≤1.0	Future $\Omega_{arag\text{-}pH}$ frequency ≤1.7	Future $\Omega_{arag\text{-}pH}$ frequency ≤1.0
Fogarty Creek	FC	44.84°N, 124.06°W	2011	2013	18.1%	7.71	7.54	7.99	0.022	49810	48.9%	11.7%	35.5%	3.5%	55.8%	28.6%
Strawberry Hill	SH	44.25°N, 124.12°W	2011	2013	16.3%	7.69	7.43	8.00	0.023	42203	46.9%	10.8%	30.9%	5.0%	52.9%	23.4%
Cape Arago	CA	43.31°N, 124.40°W	2013	2013	12.7%	7.70	7.44	8.03	0.023	8451	37.3%	9.3%	24.8%	4.0%	45.5%	18.5%
Cape Blanco	CB	42.84°N, 124.57°W	2012	2013	7.1%	7.72	7.56	8.01	0.017	19937	47.4%	3.6%	21.0%	0.8%	57.9%	12.7%
Cape Mendocino	CM	40.34°N, 124.36°W	2011	2013	0.3%	7.89	7.76	8.02	0.011	15398	33.6%	0.0%	3.9%	0.0%	61.1%	2.1%
Kibesilah Hill	KH	39.60°N, 123.79°W	2012	2013	0.3%	7.87	7.73	8.02	0.013	9185	44.1%	0.1%	18.9%	0.1%	74.2%	12.7%
Van Damme State Park	VD	39.28°N, 123.80°W	2011	2013	2.4%	7.82	7.58	7.98	0.012	35488	55.5%	0.4%	11.0%	0.0%	69.6%	6.5%
Bodega Head	BH	38.32°N, 123.07°W	2011	2013	5.6%	7.79	7.54	8.00	0.017	62321	42.8%	1.5%	15.5%	0.3%	57.3%	11.1%
Terrace Point	TP	36.95°N, 122.06°W	2011	2013	0.2%	7.96	7.66	8.15	0.015	47022	4.6%	0.0%	0.9%	0.0%	10.0%	0.4%
Hopkins	HP	36.62°N, 121.91°W	2011	2013	0.5%	7.91	7.60	8.12	0.018	40545	9.9%	0.0%	0.7%	0.0%	22.5%	0.4%
Soberanes	SB	36.45°N, 121.93°W	2013	2013	5.5%	7.79	7.50	7.97	0.016	23534	52.3%	0.9%	13.8%	0.3%	68.5%	8.2%
Lompoc Landing	LL	34.72°N, 120.61°W	2011	2011	0.7%	7.87	7.77	8.08	0.017	2877	23.5%	0.0%	2.5%	0.0%	34.5%	0.9%

Table S1. Coordinates, deployment time windows, and summary statistics of pH and $\Omega_{arag\text{-}pH}$ from the intertidal OA observing network.

Additional details on calculation of $\boldsymbol{\Omega}_{arag\text{-}pH}$

For a given pH value, possible solutions to $\boldsymbol{\Omega}_{arag}$ can vary widely with S, T, and A_T, and we explored the effects of uncertainties in these parameters. Salinity can influence calculated values of $\boldsymbol{\Omega}_{arag}$ (equation 1) through its effects on the solubility product constant (K_{sp}) (equation 2), $[Ca^{++}]$ (equation 3) and carbon speciation (Dickson 2010).

$$\boldsymbol{\Omega}_{arag} = [CO_3^{2-}] * [Ca^{2+}] / [K_{sp\text{-}aragonite}] \quad \text{(Equation 1)}$$

$$\text{Log } K_{sp\text{-}aragonite} = -171.945 - 0.077993T + 2903.293/T + 71.595\log(T) + (-0.068393 + 0.0017276T + 88.135/T)S^{0.5} - 0.10018S + 0.0059415S^{1.5} \quad \text{(Equation 2)}$$

$$[Ca^{2+}] = 0.0102821 * (S/35) \quad \text{(Equation 3)}$$

At S=33, T=10°C, a change in S by 1 (approximately 25% of the observed dynamic range in our system) results in a change in $[Ca^{++}]$ of 3% and K_{sp} of 0.4%. Carbon speciation constants k_1 (equation 4) and k_2 (equation 5) vary as a function of S and T. Salinity has a relatively minor influence on k_1 and k_2, as a shift in S by 1 unit translates into 0.9 and 2.4% changes in

$$\text{Log } (k_1/k°) = -3633.86/T + 61.2172 - 9.67770 \ln(T) + 0.011555S - 0.0001152S^2 \quad \text{(Equation 4)}$$

$$\text{Log } (k_2/k°) = -471.78/T + 25.9290 + 3.16967 \ln(T) + 0.01781S - 0.0001122S^2 \quad \text{(Equation 5)}$$

$$k° = 1 \text{ mol kg}^{-1} \quad \text{(Equation 6)}$$

k_1 and k_2, respectively, under those same T and S conditions. At pH = 8, DIC = 2200, this translates into a change in $[CO_3^{2-}]$ of 2.3%. Collectively, the uncertainty associated with the use of a mean S is on the order of +/- 0.04 units of $\boldsymbol{\Omega}_{arag}$ for every 1 unit deviation in S. Temperature influences $\boldsymbol{\Omega}_{arag}$ through its effects on K_{sp} and carbon speciation. For every 3°C change (approximately 25% of the observed dynamic range), K_{sp} shifts by 0.6%. Temperature has a much larger impact on $\boldsymbol{\Omega}_{arag}$ via its influence on k_1 and k_2, which change by 7% and 12%, respectively, with a 3°C shift. This results in a change in $\boldsymbol{\Omega}_{arag}$ by as much as 15% depending on initial T. The importance of T in constraining $\boldsymbol{\Omega}_{arag\text{-}pH}$ can be seen in Fig S2 where solutions for $\boldsymbol{\Omega}_{arag\text{-}pH}$ narrow considerably if T is known.

Error in measurement of *in-situ* T is an additional source of uncertainty. We quantified drift in sensor T measurement by cross-calibrating a subset of sensors that were previously calibrated against factory-calibrated Seabird SBE-37 temperature and conductivity sensors. Over a 6 month period, cross-calibration at monthly intervals identified no directional drift between sensors with net between sensor fluctuations that ranged from 0.001 to 0.059°C. Error in T can affect our estimates of $\boldsymbol{\Omega}_{arag\text{-}pH}$ through effects of T on calculation of pH from sensor voltage and through the effects of T on pH and $\boldsymbol{\Omega}_{arag}$. Observed range in sensor T error translates into an error in pH calculation from sensor voltage by a maximum of 0.00069 units, well outside the precision of

CRM-based calibrations employed and sensor performance. We then applied the effects of sensor T error to our calculation of $\mathbf{\Omega}_{arag}$. The net effects of the observed range in T error translate into a maximum of 0.5% error in $\mathbf{\Omega}_{arag}$. Error in measurement of S *in-situ* represents another source of uncertainty. Salinity measurements varied in precision among the research groups, depending on whether samples are analyzed as bottle samples on high precision laboratory salinometers (Autosal Guideline Instruments) or by lower precision field sensors (Yellow Springs Instrument -YSI conductivity meters). In repeated cross-calibrations against factory-calibrated Seabird SBE-37 temperature and conductivity sensors and YSI sensors, a maximum error in measurement of S of 2.7% (as residuals from calibration line) was encountered. At S of 33, this translates into a maximum error of 0.9, a value that translates into a maximum error of +/- 0.04 units of $\mathbf{\Omega}_{arag}$. We note that this is a maximum error and because of non-linearities in the carbonate system, this maximal error is suppressed under more acidified conditions, declining to +/- 0.009 units of $\mathbf{\Omega}_{arag}$ when $\mathbf{\Omega}_{arag}$ approaches minimum values for our system. Error associated with pH measurement *in-situ* represents another source of uncertainty. Because sensors were calibrated against CRM or CRM-based spectrophotometric measurements, we consider sensor drift between intervals of calibration to the key source of uncertainty. We used pre- and post- calibration values as a measure of the uncertainty associated with sensor drift. Across deployments, mean drift ($\bar{x}$ = -0.002, 95% C.I. = 0.017 pH units,) did not differ significantly from zero. Mean absolute drift was 0.032, +/- 0.037 s.d.. This translates into an error of up to +/- 0.34 in estimate of $\mathbf{\Omega}_{arag}$ at the upper range limits of pH observed in our study. However, because of the nonlinearities in the carbonate system, the effects of instrument drift translates into error of +/- 0.08 when $\mathbf{\Omega}_{arag}$ becomes corrosive, declining further to +/- 0.04 at the lower range limits of exposure.

Uncertainty in A_T is a remaining unknown in estimating $\mathbf{\Omega}_{arag}$ from pH. Our analysis suggests that accurate $\mathbf{\Omega}_{arag\text{-}pH}$ values require the collection of discrete bottle samples so that a mean A_T can be determined. With an estimated mean A_T, differences between $\mathbf{\Omega}_{arag\text{-}pH}$ and $\mathbf{\Omega}_{arag}$ will reflect deviations in the sample A_T from mean A_T. This error varies as a function of $\mathbf{\Omega}_{arag}$ with increasing variance at high $\mathbf{\Omega}_{arag}$ (Fig S5). Because the buffer factor for $\mathbf{\Omega}_{arag}$ with respect to changes in A_T reaches a minimum as DIC approaches A_T (Egleston et al. 2010), one expectation would be for differences between $\mathbf{\Omega}_{arag\text{-}pH}$ and $\mathbf{\Omega}_{arag}$ to increase at low values of $\mathbf{\Omega}_{arag}$ where DIC $\approx A_T$. At this point, $[CO_3^{2-}]$ is very small and small changes in A_T can result in large relative changes in $[CO_3^{2-}]$. Such changes are, however, small in the absolute sense, as a 20% change in $[CO_3^{2-}]$ when DIC and AT =2200 is +/- 9 μmol kg^{-1}. We estimate that at $\mathbf{\Omega}_{arag}$ between 2 and 4, error from uncertainty in A_T is approximately 0.2 for every 100 μmol kg^{-1} of A_T (Fig S5). Between $\mathbf{\Omega}_{arag}$ values of 2 and 1, this error diminishes to 0.1 for every 100 μmol kg^{-1} of A_T. Below $\mathbf{\Omega}_{arag}$ values of 1, this error is reduced even further to 0.05 for every 100 μmol kg^{-1} of A_T. The realized deviations from $\mathbf{\Omega}_{arag}$ will increase considerably in systems such as estuaries where A_T can vary widely. Because A_T has a relatively narrow window of variability (mean = 2203, s.d. = ±69) in our study, we estimate maximum deviations in $\mathbf{\Omega}_{arag\text{-}pH}$ from $\mathbf{\Omega}_{arag}$ to be 0.15 in our system. While full determination of carbon system parameters provide data of highest resolution and are often critical for studies of ocean carbon inventories and fluxes, our analyses suggest that where full determination is not possible, $\mathbf{\Omega}_{arag\text{-}pH}$ can serve as a robust proxy for $\mathbf{\Omega}_{arag}$.

Density-based estimates of anthropogenic DIC reflect the fundamental first order relationship between density, the time of last ventilation, and the corresponding levels of DIC_{ant} at

equilibration with the atmosphere. A ventilation age older than the mean yields an overestimate of the current effects of anthropogenic CO_2, but translates into greater future declines. If ventilation age is underestimated, future declines in $\mathbf{\Omega}_{arag}$ will be less than projected but the effects of DIC_{ant} would contribute to a larger portion of undersaturation events than the system faces currently. We note that because the increase in DIC_{ant} is set by differences in equilibration at 280 and 400 ppm, the expected amplitude of change from pre-industrial conditions is unaffected by uncertainties in ventilation age. We further explored the effects of uncertainties in our estimate of DIC_{ant} by examining the effects of subtracting DIC_{ant} across a window of ± 3 standard deviations. For each site (Fig 5A-C, Table S1), the observed cumulative distributions of $\mathbf{\Omega}_{arag}$ sit outside the 3 S.D. window for our estimates of pre-industrial $\mathbf{\Omega}_{arag}$.

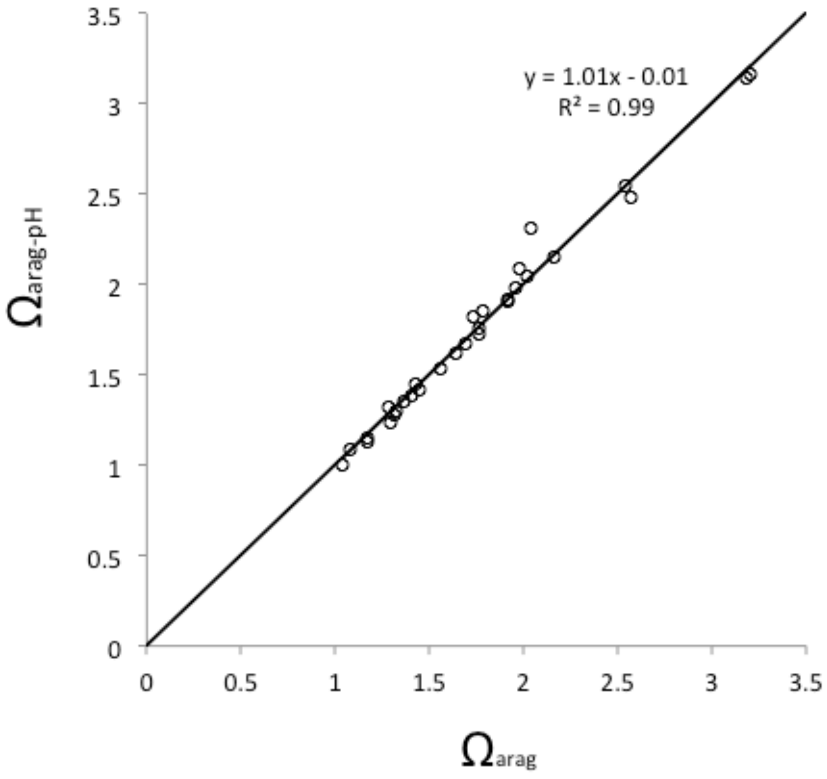

Fig S1. Ω_{arag} for discrete surf-zone samples calculated from 2 measured carbon parameters vs. $\Omega_{arag\text{-}pH}$ modeled using pH and an assumed A_T of 2200 mmol kg^{-1}.

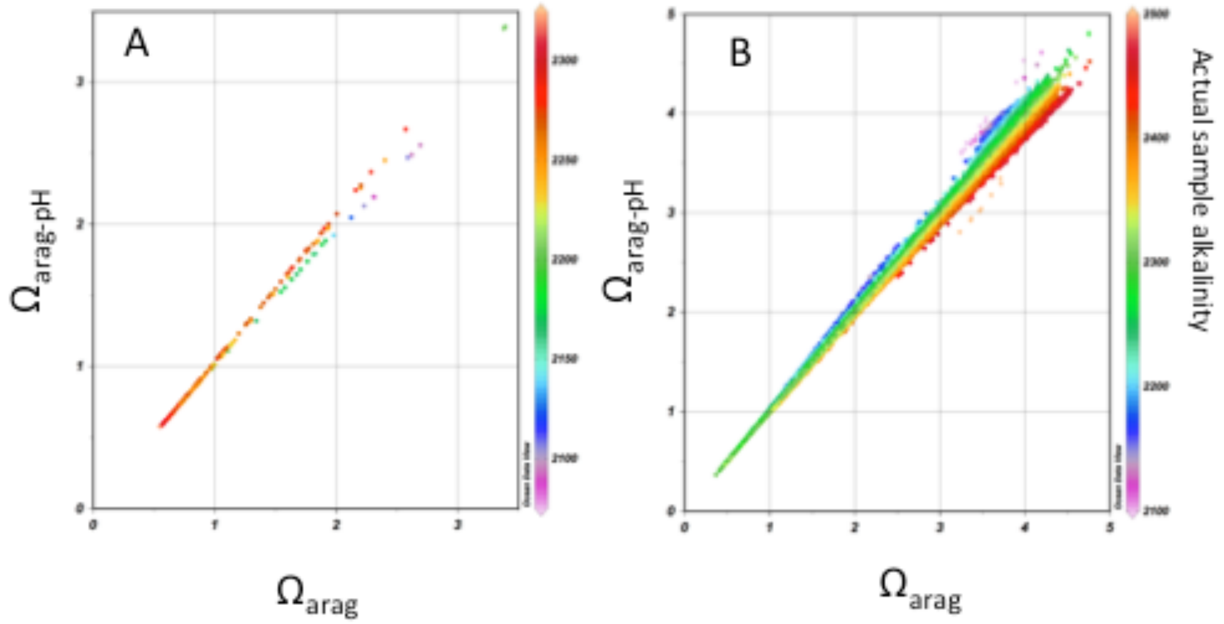

Fig S2. Estimates of $\Omega_{arag\text{-}pH}$ from pH and assigned mean A_T (2200 for NACP, 2300 for GLODAP) vs. Ω_{arag} calculated from pH and measured alkalinity for the 2007 North American Carbon Program (NACP) West Coast Cruise (A) and the GLODAP global ocean (B) databases.

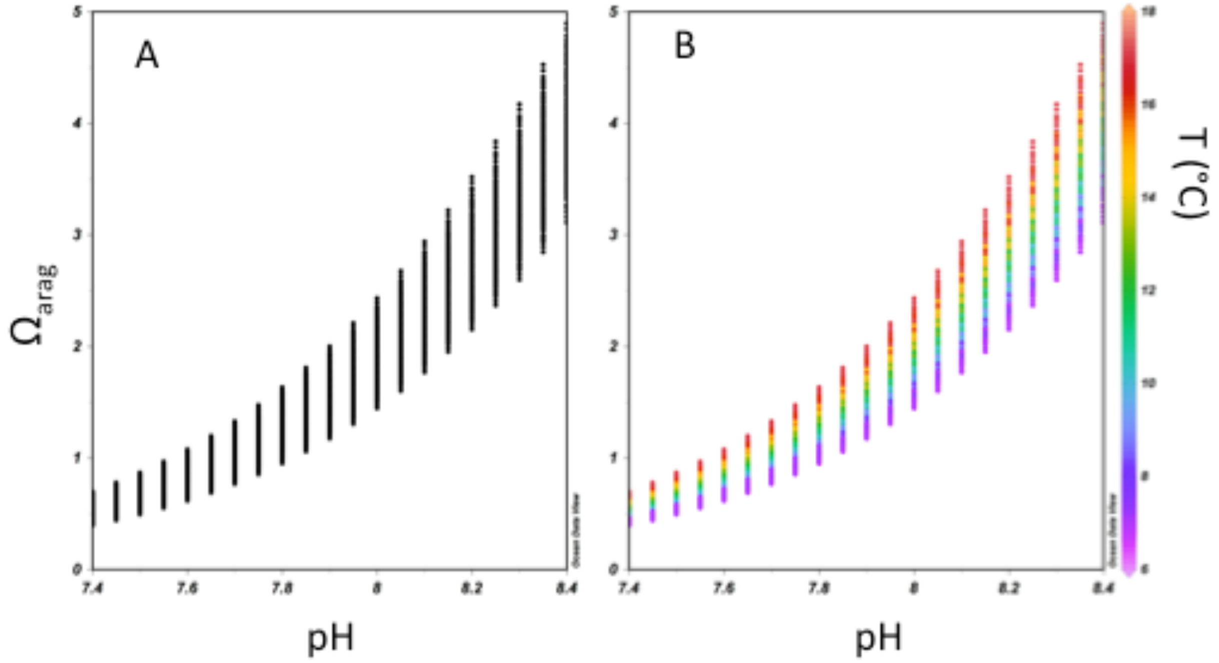

Fig S3. Potential values of Ω_{arag} as a function of pH for the range of T, S, and A_T encountered in our system where T is unknown over a range of 8 to 18°C (A), and where T is known (B).

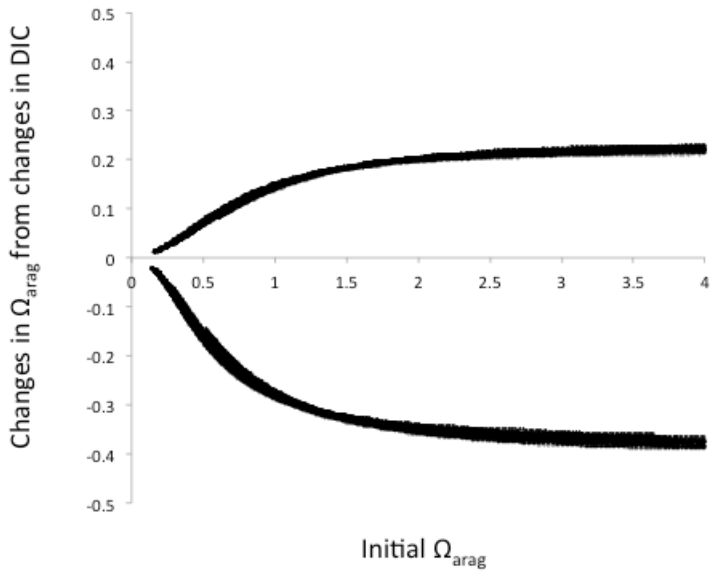

Fig S4. Simulations of Ω_{arag} changes from the subtraction of 37 μmol kg^{-1} of DIC (lower panel) and addition of 22 μmol kg^{-1} of DIC (upper panel) as a function of initial Ω_{arag} across conditions of T = 8, 18, S = 32-34, TA = 2100-2300, DIC = 1700-2350.

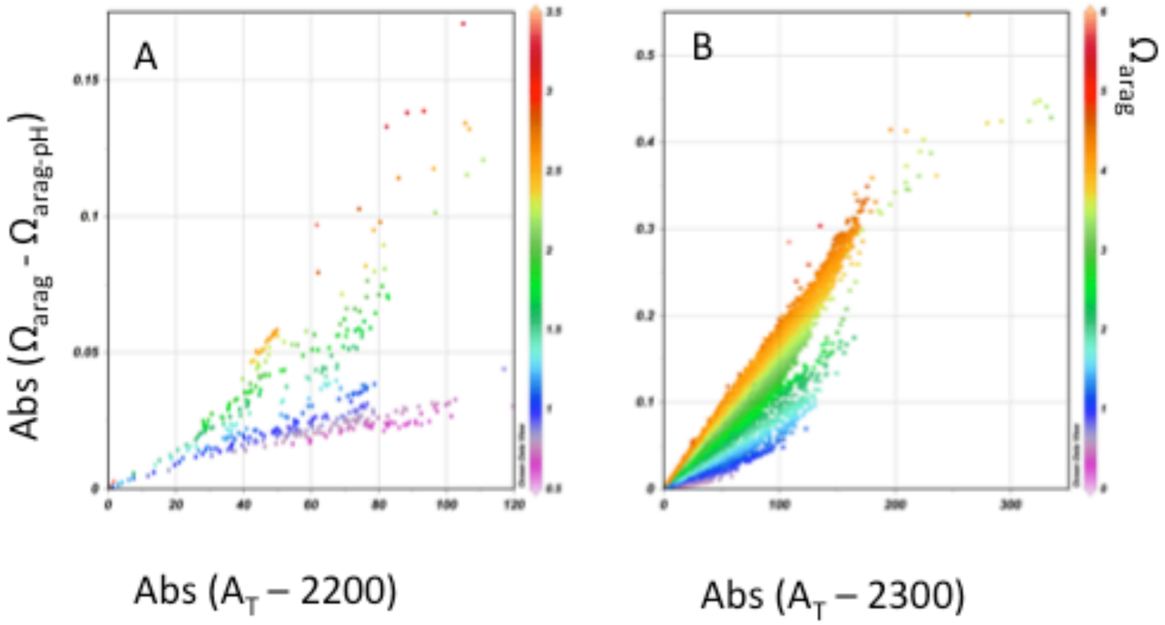

Fig S5. Error in performance of $\Omega_{arag\text{-}pH}$ (as absolute difference from Ω_{arag}) in relation to uncertainty in A_T (as the absolute difference in assigned mean A_T and measured A_T) for the NACP (A) and GLODAP (B) datasets used in Fig S5 Color scale denotes Ω_{arag} and illustrates the reduced error in $\Omega_{arag\text{-}pH}$ when Ω_{arag} is low.

Stability of results with respect to choice of summary statistics on pH and Ω_{arag} exposure serverity.

In Fig 1, we used the lower 5th percentile of pH values as a metric of exposure severity. To evaluate the stability of the reported spatial pattern to percentile choice, we've plotted the latitudinal pattern of exposure as indexed by other percentiles at 5% increments for the lower quartile values (FigS6). The latitude vs. percentile curves are all highly congruent with each other, suggesting that our finding of spatial patterning in exposure is a robust feature of the system. The stability of our results also holds for Ω_{arag} (Fig S7). In Fig 4, we've plotted ship-based offshore near-bottom pH vs. lower 5th percentile pH from intertidal stations. To evaluate the effects of using a lower 5th percentile over other percentile thresholds, we performed the same regression for other percentiles at 5% increment up to the 95th percentile. The R^2 of regressions reach a maximum (0.87) at the 35th percentile (Fig S8a) and suggests that Fig 4 represents a conservative test of the strength of the relationship between offshore and intertidal pH exposure. R^2 values decline after the 35th percentile and regression slopes are not significant after the 55th percentile (i.e. for higher pH encountered in the intertidal). This is expected as ship-based near-bottom measures represent the local minima in source pH and not a mean value, and because low intertidal pH values arise from upwelling events that bring cold, low pH water from depth over the shelf to the shore (Fig 3). The connection between bottom shelf waters and the shore is further evident in a cross-shelf section (Fig S9) from the NOAA West Coast Ocean Acidification WCOA2011[34] from 44.20°N that is combined with intertidal measurement from the SH site (44.25°N). The cross-shelf section took place during a period (Aug 18th 2011) of upwelling favorable winds that resulted in the shoreward bottom flows and shoaling of density, pH, and Ω_{arag} layers (Fig S9a). We do note that while the relationship is quite strong (max R^2 of 0.87, $p<0.005$), additional observations will be important for fully evaluating the persistence of the offshore shelf patterns in bottom pH, and to further improve the predictive precision and geographic generality of cross-shelf coupling in low pH exposure.

Whereas upwelling circulation connects offshore waters from depth to the shore to influence the severity of coastal low pH exposure (Fig S8a), we also explored the strength of shelf to shore coupling in describing exposure to high pH values. During wind relaxation or downwelling-favorable event, offshore surface waters flow shoreward bringing warm and relatively high pH values to the coast. Surface pH values from the NOAA shelf stations were significantly predictive of how the upper range of pH values (80th to 95th percentile) varied across the 7 stations of the 2011 intertidal observing network (Fig S8b). We note however that the strength of the relationship between offshore bottom measurements and low pH exposure, and that for offshore surface measurement and high pH exposure do not appear to be asymmetrical. Offshore surface values significantly account for a narrower range (80th to 95th percentile vs 5th to 55th percentile), lower portion of the variation (max R^2 of 0.68 vs. 0.87), and lower statistical significance (min p-value of 0.02 vs. 0.002 for test of slope) in upper pH exposure relative to the performance of deep values in accounting for lower pH exposure (Fig 8a,b). The lower strength of surface to shore coupling point to additional factors such as nearshore productivity, and heterogeneity in offshore source water properties that can modulate the cross-shelf pH connectivity.

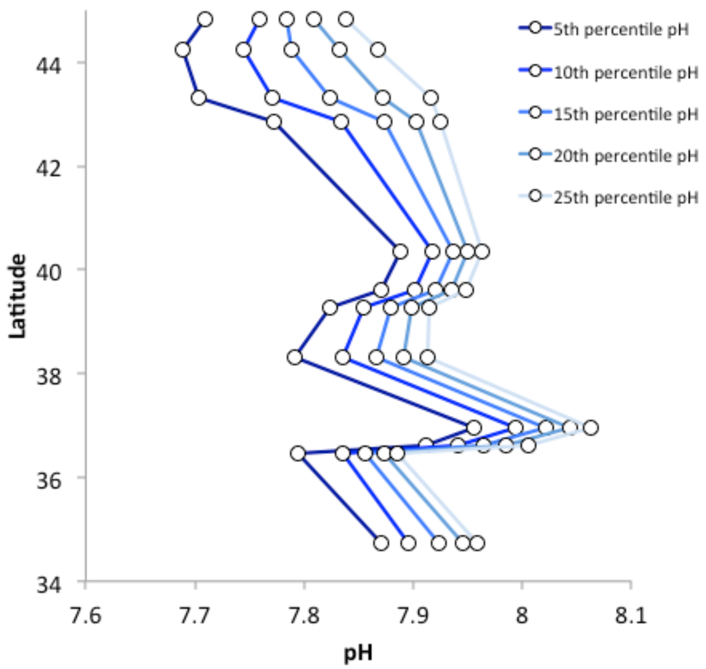

Fig S6. Consistency in the spatial pattern of intertidal pH across different metrics of low pH exposure from lower 5th to 25th percentile.

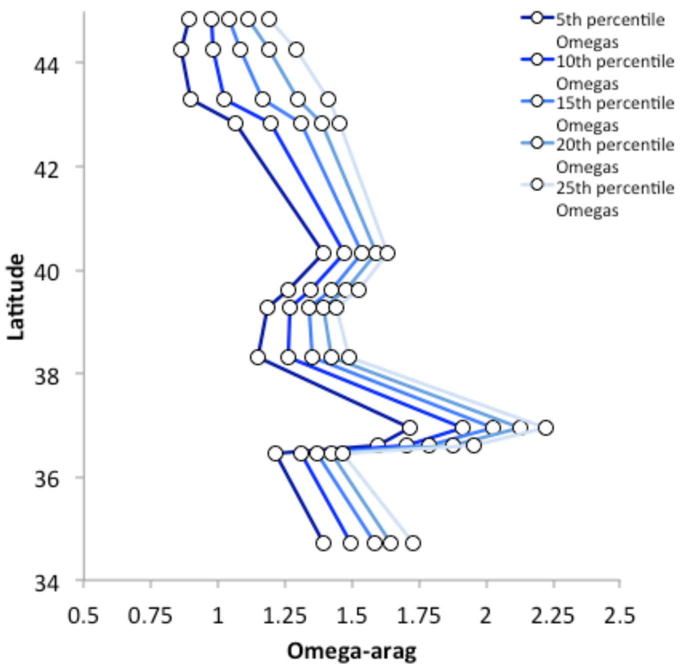

Fig S7. Consistency in the spatial pattern of intertidal $\mathbf{\Omega}_{\text{arag-pH}}$ across different metrics of low $\mathbf{\Omega}_{\text{arag-pH}}$ exposure from lower 5th to 25th percentile.

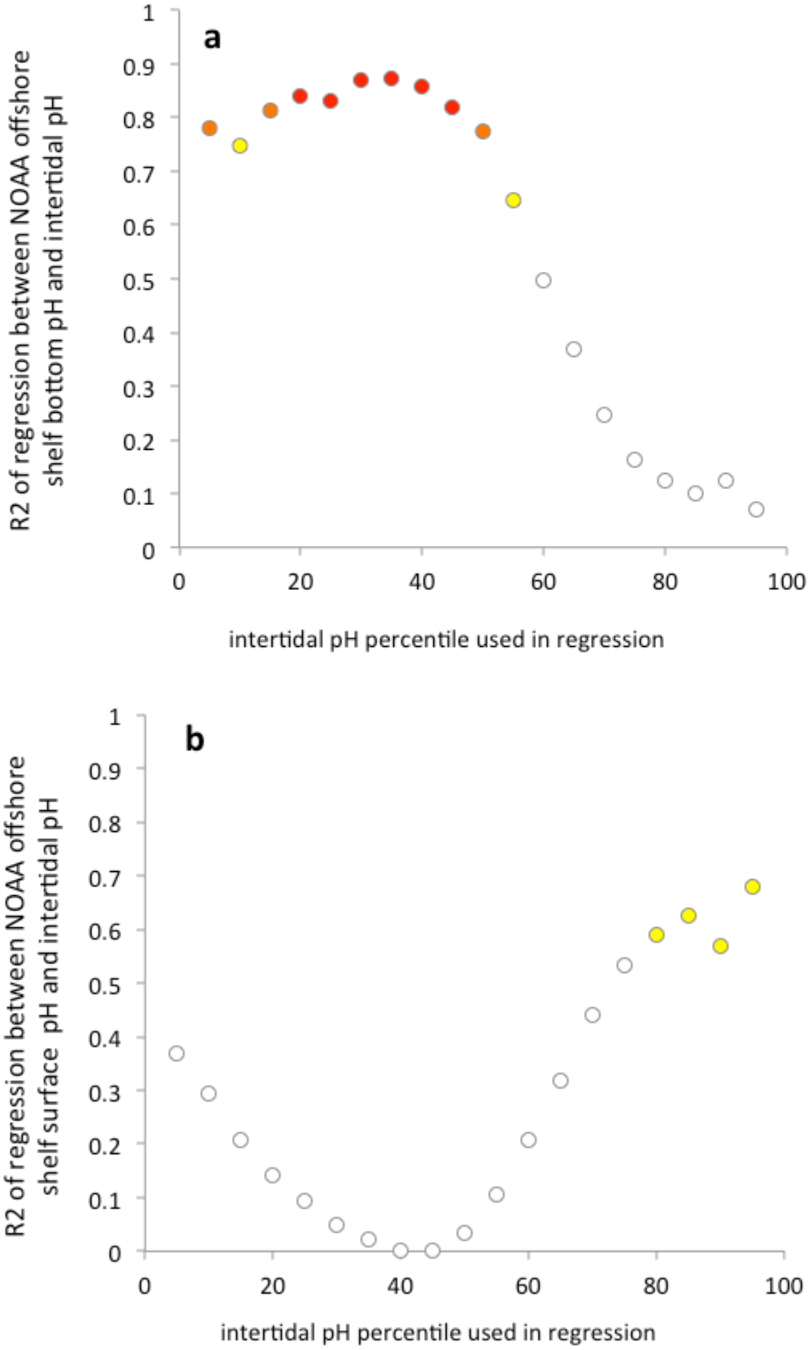

Fig S8. (a) Effects of percentile choice on the coefficient of determination (R^2) value for regressions between intertidal and offshore (ship-based) pH measurements from depth. (b) same as (a) but with offshore surface value as independent variable in regressions. Color-filled symbols reflect significance test of slopes for p-values of <.05 (yellow), <.01 (orange), <.005 (red). Open symbols are not significant.

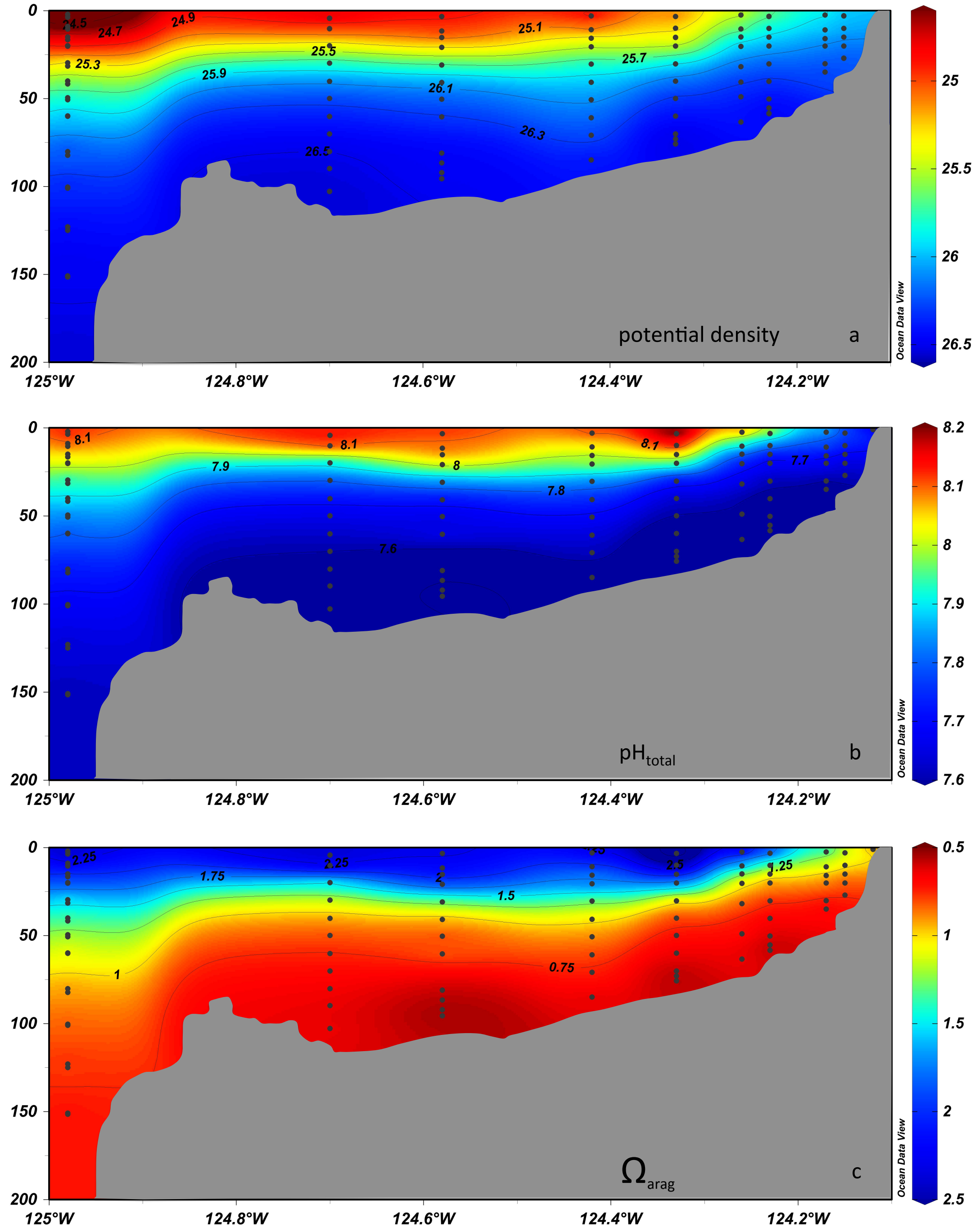

Fig S8. Cross-shelf section of (a) density, (b) pH_{total}, (c) Ω_{arag} from the NOAA WCOAH cruise across 44.20°N on August 18th 2011 illustrating the hydrographic connectivity between the shelf and the nearshore. Intertidal values for pH and $\Omega_{arag\text{-}pH}$ from sensor at 44.25°N (SH) are also plotted in b and c as the 1st coastal sample point.

Generality of inner-shelf and intertidal pH coupling

To further examine the generality of the coupling between inner-shelf and intertidal pH highlighted in Fig 3B, we've plotted two additional time-series where mooring-based sensors were deployed directly offshore of intertidal sensors at Bodega Head (38.32°N, Fig S10.) and Terrace Point (36.95°N, Fig S11) in 2013. Both moorings were deployed in 20m depth with sensors positioned near-surface. A Durafet®-based Seafet pH sensor was deployed at Bodega Head (at 15min intervals) while Terrace Point consisted of the same custom Durafet®-based pH sensor (at 10 min intervals) as used in the intertidal. The longest time windows where deployments of both moored and intertidal sensors were successful are presented here. Calibration procedures were the same as described in the methods for other sensors in the network. Calibration values were however not available for the successful deployment interval of the Bodega Head mooring sensor. Raw, uncalibrated values are shown here. Accordingly that record is used only for the purpose of illustrating the temporal coherence of pH variability across nearshore habitats. As in Fig 3B, the intertidal (red) and mooring (blue) time-series were low pass filtered (40hr window LOESS filter) (in bold lines). Unfiltered time-series are depicted in the background. For both sites, inner-shelf and intertidal pH fluctuated in concert. There are periods of decoupling but the correspondence in their responses to event-scale variability is similar to that observed in Central Oregon at 44.25°N.

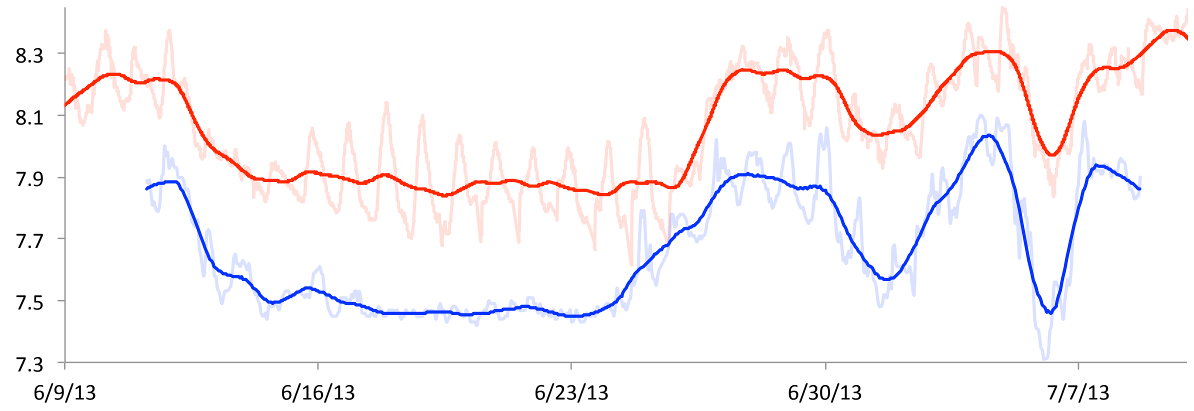

Fig S10. Coupled pH time-series from inner-shelf an intertidal sensors at Bodega Head (38.32°N) in 2013. Intertidal pH (red) data are calibrated and quality-controlled, mooring pH (blue) are raw values and shown here for the purpose of illustrating temporal variability.

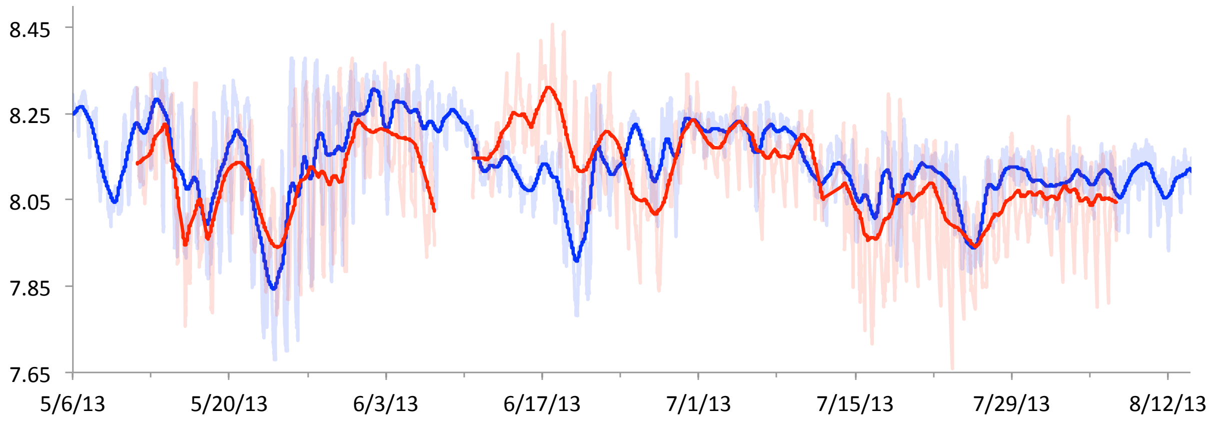

Fig S11. Coupled pH time-series from inner-shelf an intertidal sensors at Terrace Point (36.95°N) in 2013. Intertidal pH (red) and mooring pH (blue) data are depicted.

Supplementary Materials References

Dickson, A. G., in *Guide to best practices for ocean acidification research and data reporting,* U. Riebesell, V. J. Fabry, L. Hansson, J.-P. Gattuso, Eds. (Publications Office of the European Union, Luxembourg 2010).

Egleston, E. S., Sabine, C. L., & Morel, F. M. 2010. Revelle revisited: Buffer factors that quantify the response of ocean chemistry to changes in DIC and alkalinity. *Global Biogeochem. Cycles* **24**, B003407 (2010).
